# Risk factors and titers of COVID-19 infection in a longitudinal statewide seroepidemiology cohort

**DOI:** 10.1186/s12879-023-08670-6

**Published:** 2023-10-11

**Authors:** Elizabeth T. Rogawski McQuade, Lea Becker, Suzanne E. Stroup, Fauzia Khan, Bhruga Shah, John Brush, Gay Goldsmith, Rebecca Mullin, Danielle Guilliams, Christopher deFilippi, Kathleen Barackman, Andrea B. Mohr, Francis Farrell, Gonzalo Bearman, Lilian Peake, Eric R. Houpt

**Affiliations:** 1 Division of Infectious Diseases and International Health, University of Virginia, Charlottesville, VA USA; 2 Department of Epidemiology, Emory University, Atlanta, GA USA; 3 Research & Clinical Trial Analytics Team, University of Virginia, Charlottesville, VA USA; 4 Inova Heart and Vascular Institute, Inova Health System, Falls Church, VA USA; 5 Office of Clinical Research, Sentara Healthcare, Norfolk, VA USA; 6 Division of Infectious Diseases, Virginia Commonwealth University, Richmond, VA USA; 7 Carilion Clinic, Roanoke, VA USA; 8 Virginia Department of Health, Richmond, VA USA

**Keywords:** COVID-19, SARS-CoV-2, Seroepidemiology, Nucleocapsid, Spike, Vaccine, Risk factors

## Abstract

**Background:**

Virginia is a large state in the USA, yet it remains unclear what percentage of the population has had natural COVID-19 infection and whether risk factors for infection have changed over time.

**Methods:**

Using a longitudinal cohort, from December 2021-July 2022 we performed follow up serology and a questionnaire on 784 individuals from across Virginia who had previously participated in a statewide COVID-19 seroepidemiology study in 2020. Children were also invited to participate and an additional 62 children also completed the study. Serology was performed using Roche nucleocapsid and spike serological assays.

**Results:**

The majority of participants were white (78.6%), over 50 years old (60.9%), and reported having received COVID-19 vaccine (93.4%). 28.6% had evidence of prior COVID-19 infection (nucleocapsid positive). Reweighted by region, age, and sex to match the Virginia census data, the seroprevalence of nucleocapsid antibodies was estimated to be 30.6% (95% CI: 24.7, 36.6). We estimated that 25–53% of COVID-19 infections were asymptomatic. Infection rates were lower in individuals > 60 years old and were higher in Blacks and Hispanics. Infection rates were also higher in those without health insurance, in those with greater numbers of household children, and in those that reported a close contact or having undergone quarantine for COVID-19. Participants from Southwest Virginia had lower seropositivity (16.2%, 95% CI 6.5, 26.0) than other geographic regions. Boosted vaccinees had lower infection rates than non-boosted vaccinees. Frequenting indoor bars was a risk factor for infection, while frequently wearing an N95 mask was protective, though the estimates of association were imprecise. Infection rates were higher in children than adults (56.5% vs. 28.6%). Infection in the parent was a risk factor for child infection. Spike antibody levels declined with time since last vaccination, particularly in those that were vaccinated but not previously infected. Neutralizing antibody positivity was high (97–99%) for wild type, alpha, beta, gamma, delta, and omicron variants. Neutralizing antibody levels were higher in the follow-up survey compared to the first survey in 2020 and among individuals with evidence of natural infection compared to those without.

**Conclusions:**

In this longitudinal statewide cohort we observed a lower-than-expected COVID-19 infection rate as of August 2022. Boosted vaccinees had lower infection rates. Children had higher infection rates and infections tracked within households. Previously identified demographic risk factors for infection tended to persist. Even after the omicron peak, a large number of Virginians remain uninfected with COVID-19, underscoring the need for ongoing vaccination strategies.

**Supplementary Information:**

The online version contains supplementary material available at 10.1186/s12879-023-08670-6.

## Background

Approximately 3 years into the COVID-19 pandemic, case-based surveillance for infection remains limited by large numbers of asymptomatic, undiagnosed, and unreported cases. Serological testing on a population level therefore remains useful to document COVID-19 incidence and population level immunity. Current serodiagnostics detect SARS-CoV-2 nucleocapsid antibodies, which indicate prior natural infection, and SARS-CoV-2 spike antibodies, which can indicate either prior infection or vaccination with spike protein-based vaccines.

A number of seroprevalence studies have been performed in the United States. Some include testing of convenience residual blood samples from commercial laboratories [1]. The most recent data from Virginia from this source indicate an approximately 45% nucleocapsid antibody prevalence as of February 2022. Blood donation surveys also exist and the most recent data from September 2022 indicates that 64.4% had nucleocapsid antibodies [2]. These studies generally lack patient demographic and clinical information so one cannot assess individual-level risk factors or behaviors that are associated with infection.

We previously performed a statewide cross-sectional surveillance study of 4675 adult outpatients presenting for non-COVID-19 related health care appointments from June to August 2020 [3]. This is a uniquely representative cohort because enrollment was stratified to match state and regional age, race, and ethnicity demographics. This was early in the pandemic and the weighted seroprevalence for nucleocapsid antibodies was only 2.4%. In this study, approximately 2 years later, we re-contacted these individuals to assess updated infection rates, re-ascertain risk factors, and quantify population level vaccine and infection-induced immunity. We also performed neutralizing antibody testing for wild-type, alpha, beta, gamma, delta, and omicron variants.

## Methods

### Study design

Adults who were enrolled in a statewide cross-sectional surveillance study [3] from June to August 2020 and who consented to be contacted for follow-up (n = 4030) were eligible to participate. The previous study enrolled individuals presenting for scheduled outpatient clinic or outpatient laboratory appointments, who were not being evaluated for COVID-19, at 5 geographically diverse health system sites: the University of Virginia Health System in the Northwest, INOVA Health system in the North, Sentara Healthcare in the Southeast, Virginia Commonwealth University in the Central region, and Carilion Clinic in the Southwest. Enrollment was stratified and capped to meet the age, racial, and ethnic demographic profile of the region. Participants received invitations to enroll in this follow-up study by US mail or email and were followed up by telephone. We attempted to contact each participant at least 3 times until enrolled, declined, or deemed unable to reach. Full participation in the follow-up study required completing a questionnaire survey by phone, mail, and/or electronically, then traveling to a designated study site to complete a study-specific blood draw for SARS-CoV-2 serologic testing. A subset of participants from the Northwest region were also offered the option to use a home collection blood draw device (23% of those subjects used this option). Verbal or electronic consent was obtained for each participant. Participants received $25 compensation for study completion. The University of Virginia Institutional Review Board exempted approval for the study, since it was deemed to not constitute human subjects research but to constitute public health surveillance by the Virginia Department of Health according to 45 CFR 46.102. All surveillance methods were performed in accordance with state guidance. Informed consent was obtained from all subjects and/or their legal guardian(s).

### Serology

Serum or plasma was collected and tested using Elecsys Anti-SARS-CoV-2 assay (Roche, Mannheim, Germany) for nucleocapsid antibodies and Elecsys Anti-SARS-COV-2 S assay (Roche, Mannheim, Germany) for semi-quantitative spike antibodies according to the manufacturer’s instructions. If spike antibodies exceeded the limit of detection (> 250 U/mL) if sufficient volume they underwent serial dilution to assess quantities up to > 2500 U/mL.

### Neutralizing antibody testing

Residual serum or plasma was tested for neutralizing antibodies to SARS-CoV2 wild type, α B.1.1.7, β B.1.351, γ P.1, δ B.1.617.2, and ο B.1.1.529 using the ProcartaPlex Human SARS-CoV2 Variants Neutralizing Antibody Panel (Invitrogen, Vienna, Austria) according to the manufacturer’s instructions. We tested samples that were nucleocapsid positive from the first survey round and all available samples that were spike positive (a subset of which were also nucleocapsid positive) from the second survey round. Per the manufacturer’s instructions, a neutralization rate of ≥ 20% is considered positive for SARS-CoV-2 neutralizing antibodies, while < 20% is considered negative.

### Statistical analysis

We estimated the crude prevalence of seropositivity for the nucleocapsid and spike SARS-CoV-2 antigens by subgroups of interest and also reweighted seroprevalence by region, age, and sex to match regional population estimates for age and sex obtained from the University of Virginia Weldon Cooper Center per the population on July 1, 2019 [3]. Given the fewer participants in this follow-up study, we were not able to also reweight to race, ethnicity, and insurance status as in the first survey. As previously, we used an iterative proportional fitting procedure (raking) to estimate sampling weights based on age categorized into 18–39, 40–59, 60 + years. We used the *survey* package in R version 4.1.0 (http://www.R-project.org/) to estimate the raked weights to match the cross-classified distribution of age and sex at the regional level. As done previously [3], we also corrected prevalence estimates to account for imperfect sensitivity and specificity of the serology assays using the formula: *P*_corrected_ = (*P* + *Sp* − 1)/(*Se* + *Sp* − 1),

where *P* is the weighted prevalence, *Se* is the sensitivity of the assay, and *Sp* is the specificity of the assay. For the spike assay, based on previous studies the sensitivity was assumed to be 99.95% (95% CI: 99.87, 99.99), and specificity was assumed to be 97.92% (95% CI: 95.21, 99.32) [4]. For the nucelocapsid assay, sensitivity was assumed to be 93.61% (95% CI: 89.51, 96.46), and specificity was assumed to be 99.85% (95% CI: 99.75, 99.92) [5]. We used the delta method to incorporate variability around these estimates into the 95% confidence intervals of the corrected prevalence estimates. Seroprevalence was estimated in subgroups defined by region, age, sex, race, ethnicity, insurance status, high risk health condition, report of COVID-19-like illness, and report of COVID-19 positive test result. Analyses were conducted separately for adult and pediatric participants. Seropositivity among pediatric participants was not reweighted to census data due to small numbers. We identified risk factors for SARS-CoV-2 nucleocapsid seropositivity in the adult participants using logistic regression. We assessed sociodemographics, living environment, working environment, contact with someone with COVID-19, frequency of attending public places, mask wearing behavior, vaccination history, and self-report of illness. Multivariable regression models did not converge due to small numbers in several significant risk factor categories and are therefore not reported. Among pediatric participants, we estimated the associations between vaccination and nucleocapsid seropositivity, and between nucleocapsid seropositivity in the child and in the adult participant living in the same household. We compared SARS-CoV-2 spike seropositivity with report of vaccination and associated spike antibody quantities with time since last vaccine dose using log-binomial regression for the prevalence of spike antibody quantities above the limit of quantification. We also estimated similar associations between spike antibody quantities and age, sex, the vaccine product received, or the number of doses received using univariable log-binomial regression models. Finally, we compared antibody neutralization between naturally infected and vaccinated, and between the first and second survey using linear regression with robust variance to account for the skewed distribution of neutralizing antibodies and correlation of results for the few individuals (n = 9) who had neutralizing antibody data at both time points.

## Results

Of 4030 individuals who completed the first seroprevalence survey in June to August 2020 and consented to follow up, we restricted analyses to the 784 (20%) individuals who completed the follow-up study including a blood draw. Most of the other individuals in the cohort did not respond despite at least 3 contact attempts (n = 2241, 56%). A small proportion of individuals declined participation (n = 349, 9%) or were no longer eligible because they moved outside Virginia (n = 107, 3%) or died (n = 31, 1%), with 3 deaths reported from COVID-19. An additional 518 (13%) completed the survey but not the blood draw. Participation rates varied by site, with 38% (n = 299) of participants completing the study in the Northwest region of the state, while only 12% (n = 90) participants completed the study in the Central region.

The demographics of the individuals who completed the follow-up study were enumerated and compared with the first survey (Table S1). Participants were majority white (n = 616, 78.6%), non-Hispanic (n = 749, 95.5%), and 50 years of age or older (n = 477, 60.9%). A higher proportion of participants came from these categories versus the first survey (66.3%, 91.5%, and 49.6% respectively). In addition, 732 (93.4%) reported receipt of at least one dose of a COVID-19 vaccine, 727 (92.7%) were fully vaccinated, and 604 (77.0%) were boosted. Almost half of participants reported “never” spending time indoors without a mask, and reporting indoor dining was rare.

Approximately a quarter of participants (n = 224, 28.6%) were SARS-CoV-2 nucleocapsid antibody positive, while almost all participants (n = 760, 96.9%) were SARS-CoV-2 spike antibody positive (Table S2). Nearly three-quarters (n = 504, 73.4%) of those with positive spike antibody had quantities above our limit of quantification (> 2500 U/ml). Reweighted by region, age, and sex to match the Virginia census data and corrected for diagnostic test characteristics, the seroprevalence of nucleocapsid antibodies was 33.1% (95% CI: 26.6, 39.6; Table 1).

**Table 1:** SARS-CoV-2 nucleocapsid seroprevalence across Virginia by geography and subgroup

Region/subgroup	Number of participants	Number seropositive	Raw prevalence (%)	Adjusted^d^ Prevalence (95% CI)
Geographical Region				
Central	90	31	34.4	43.6 (27.8, 59.3)
East	206	79	38.3	41.2 (31.0, 51.5)
North	81	16	19.8	31.0 (14.6, 47.5)
Northwest	297	83	27.9	31.0 (24.3, 37.8)
Southwest	108	15	13.9	16.0 (6.3, 25.7)
Virginia (Overall)	782	224	28.6	33.1 (26.6, 39.6)
Age (years)				
18–29	65	20	30.8	28.9 (4.0, 53.8)
30–39	145	50	34.5	38.7 (24.3, 53.1)
40–49	96	36	37.5	38.8 (26.4, 51.3)
50–59	184	61	33.2	32.2 (23.8, 40.6)
60–69	175	38	21.7	20.4 (13.7, 27.1)
70–79	100	15	15	13.7 (6.6, 20.8)
≥80	17	4	23.5	26.1 (1.9, 50.2)
Gender				
Female	526	162	30.8	33.8 (27.9, 39.7)
Male	256	62	24.2	32.4 (20.5, 44.2)
Race				
White	615	164	26.7	28.6 (22.9, 34.2)
African American	98	40	40.8	48.0 (28.4, 67.6)
Asian	26	5	19.2	28.2 (-6.4, 62.8)
Two or more races	12	5	41.7	31.2 (-1.5, 63.8)
Other race	31	10	32.3	60.3 (30.8, 89.7)
Ethnicity				
Hispanic	34	13	38.2	63.3 (34.3, 92.4)
non-Hispanic	748	211	28.2	31.5 (25.0, 38.0)
Health-insurance at time of survey^a^				
Medicaid^b^	75	27	36	39.3 (23.4, 55.2)
Medicare	199	41	20.6	24.6 (15.2, 34.1)
Private (employer or individual)	470	143	30.4	33.6 (25.6, 41.6)
Military, Tricare, or Veterans Administration	23	5	21.7	23.3 (2.2, 44.3)
None or uninsured	15	10	66.7	71.3 (40.7, 102.0)
High-Risk Health Condition^c^				
Yes	300	87	29	34.8 (25.7, 43.9)
No	478	136	28.5	32.7 (24.1, 41.4)

^a^ Health insurance missing for 9 individuals

^b^ Medicaid includes FAMIS, Virginia’s health insurance program for children

^c^ diabetes, lung disease (including moderate to severe asthma), a severe heart condition, kidney disease, liver disease, or immunocompromised; missing for 4 individuals

^d^ Reweighted by region, age, and sex to match regional population estimates and corrected for imperfect sensitivity and specificity of diagnostic tests

Seroprevalence varied by geographic region, was highest in Central Virginia (43.6%, 95% CI: 27.8, 59.3) and lowest in Southwest Virginia (16.0%, 95% CI: 6.3, 25.7). Seroprevalence was higher in individuals aged 30–49, among non-White and non-Asian participants, and among the uninsured and those with Medicaid insurance. Few individuals (n = 9) who were nucleocapsid seropositive at the time of the first survey were evaluated in the current study. However 7 of 9 remained nucleocapsid positive in this study.

329 individuals (42.0%) reported one or more COVID-19-like-illnesses since the prior survey and among the 229 (29.2%) who were tested for COVID-19 for at least one of these illnesses, 119 (52.0%) tested positive (Table S3). Hospitalization for COVID-19-like illnesses was reported by 18 (2.3%). Positive COVID-19 results on tests administered for other reasons (i.e., while asymptomatic) was rare (11 additional positives among 340 individuals asked about positive tests taken for non-illness reasons). Of the 130 participants that reported any prior positive COVID-19 test, 82.3% (n = 107) were nucleocapsid seropositive. Of 224 total nucleocapsid seropositive individuals, 169 reported a COVID-19-like illness since the beginning of the pandemic, such that 25% of infections could have been asymptomatic, assuming all 169 of these individuals indeed had COVID-19. On the other hand, only 105 of these 169 individuals reported a positive COVID-19 test during their illness, such that 53% of infections could have been asymptomatic, assuming all remaining COVID-19-like illnesses reported were actually COVID-negative.

Risk factors associated with nucleocapsid seropositivity were largely limited to demographic characteristics, known contact with COVID-19 positive individuals, and vaccination (Table 2).

**Table 2:** Risk factors for seropositivity

	N	Odds Ratio (95% CI)
Characteristic		Univariable model
Region		
East	206	0.91 (0.44, 1.88)
North	81	0.56 (0.21, 1.48)
Northwest	297	0.59 (0.30, 1.16)
Southwest	108	0.26 (0.10, 0.65)
Central	90	Ref.
Age		
50+	476	0.72 (0.44, 1.21)
18–49	306	Ref.
Gender		
Male	256	0.98 (0.55, 1.75)
Female	526	Ref.
Race		
African American	98	2.31 (1.05, 5.05)
Asian	26	1.02 (0.19, 5.46)
Two or more races or other race	43	2.66 (1.00, 7.10)
White	615	Ref.
Ethnicity		
Hispanic	34	3.44 (1.08, 10.98)
Non-Hispanic	748	Ref.
Health Insurance		
Medicaid^a^	66	1.23 (0.57, 2.65)
Medicare	199	0.67 (0.37, 1.23)
Military, Tricare, or Veterans Administration	23	0.62 (0.19, 2.07)
None or uninsured	15	4.48 (1.18, 17.00)
Private (employer or individual)	470	Ref.
High-risk health condition		
Yes	300	1.13 (0.66, 1.94)
No	478	Ref.
Type of Residence		
Other^b^	149	0.83 (0.39, 1.80)
Single family home	633	Ref.
Number of household adults	784	1.29 (0.96, 1.73)
Number of household children	784	1.41 (1.09, 1.83)
Worked outside the home since June-August 2020		
Yes	421	1.18 (0.64, 2.19)
No	342	Ref.
Close contact with COVID + individual		
Yes	341	2.93 (1.58, 5.43)
No	352	Ref.
Asked to quarantine for contact with COVID + individual		
Yes	123	5.07 (2.55, 10.09)
No	641	Ref.
Frequency of indoor dining		
More than once a month	277	0.90 (0.51, 1.59)
Once a month or less	482	Ref.
Frequency of visiting indoor bar		
More than once a month	56	2.27 (0.99, 5.21)
Once a month or less	700	Ref.
Time indoors in public without a mask		
More than once a month	324	1.63 (0.89, 2.99)
Once a month or less	433	Ref.
Time indoors in public with others not wearing masks		
> 50% of the time	531	1.42 (0.76, 2.62)
< 50% of the time	226	Ref.
Time outdoors in public with a mask		
> 50% of the time	368	1.16 (0.66, 2.05)
< 50% of the time	384	Ref.
Type of mask worn most often		
N95 equivalent (KN95/KF94)	177	0.54 (0.28, 1.06)
Surgical mask	319	1.04 (0.55, 1.98)
Other	286	Ref.
Received COVID-19 vaccine		
No	39	1.88 (0.66, 5.40)
Yes (at least 1 dose)	730	Ref.
COVID-19 vaccine received (dose 1)		
Johnson and Johnson	51	0.59 (0.21, 1.69)
Moderna	263	0.76 (0.39, 1.51)
No vaccine	51	1.85 (0.73, 4.70)
Pfizer	416	Ref.
Received monovalent booster vaccine dose^c^		
Yes	604	0.43 (0.24, 0.78)
No	178	Ref.
Self-report COVID-19-like illness		
Yes	328	8.39 (4.78, 14.74)
No	441	Ref.

^a^ Medicaid includes FAMIS, Virginia’s health insurance program for children

^b^ Multi-family/Apartment /Condominium, long-term care facility or other congregate setting, or other

^c^ Booster vaccine was defined as a second dose if first dose was Johnson and Johnson and a third dose if first dose was Pfizer or Moderna

The odds of seropositivity among individuals from Southwest Virginia were lower than that among individuals from other regions (OR: 0.36, 95% CI: 0.16, 0.76). The odds of seropositivity were higher among African American (OR: 2.31, 95% CI: 1.05, 5.05) and Hispanic (OR: 3.44, 95% CI: 1.08, 10.98) participants compared to White and non-Hispanic participants. The odds of seropositivity among the few participants who were uninsured were also higher than the insured (OR: 4.48, 95% CI: 1.18, 17.00). The odds of seropositivity increased by 41% (95% CI: 9, 83) for each additional child living in the household. The odds of seropositivity among tndividuals who had a close contact with a COVID-19 positive individual were almost 3 times the odds among those who did not report such a contact (OR: 2.93, 95% CI: 1.58, 5.43). The odds of seropositivity among individuals who received a COVID-19 vaccine booster were 57% (95% CI: 22, 76) lower compared to those who had not received a booster. None of the behavioral risk factors were statistically significantly associated with seropositivity. However, high frequency of visiting an indoor bar was associated with increased seroprevalence, while wearing a N95-equivalent mask was associated with lower seroprevalence.

In contrast to the nucleocapsid results, the reweighted seroprevalence of spike antibodies (97.5%, 95% CI: 96.1, 98.9) was universally high across regions and subgroups. After adjusting for imperfect sensitivity and specificity of the diagnostic assay, seroprevalence was estimated to be above 100% for almost all subgroups of interest (Table S4). Almost all individuals who reported at least one vaccine dose (n = 723/729, 99.2%) were spike seropositive. The 6 individuals who reported vaccination but were spike seronegative were between 50 and 59 years old (n = 5) and 80 years old (n = 1). All received at least 2 vaccine doses and 5 of 6 had received a booster dose. Most individuals who were spike seropositive and did not report vaccination were also nucleocapsid positive (n = 20/24), indicating natural infection. Vaccinated spike seropositive individuals had higher spike antibody quantities than unvaccinated spike seropositive individuals (74.5% of vaccinees had spike antibody quantities > 2500 U/ml vs. 10.8% of unvaccinated individuals).

Spike antibody quantities were associated with time since last vaccine dose. A lower proportion of individuals had spike quantities above 2500 U/ml as time since last vaccine dose increased (Table 3).

**Table 3:** Association between SARS-CoV-2 spike IgG antibody titers and time since last COVID-19 vaccine dose

Time since last vaccine dose	Number	N (%) with spike > 2500 U/ml	Prevalence ratio (95% CI)
< 3 months	155	135 (87.1%)	1
3–5 months	304	233 (76.6%)	0.88 (0.81, 0.96)
6–8 months	92	68 (73.9%)	0.85 (0.74, 0.97)
9–11 months	55	28 (50.9%)	0.58 (0.45, 0.76)
≥ 12 months	34	16 (47.1%)	0.54 (0.38, 0.78)

For example, the prevalence of spike levels above 2500 U/ml was 46% lower (95% CI: 22, 62) among individuals vaccinated more than 12 months ago compared to those who received their last dose within the previous 3 months. This temporal association was stronger in spike positive/nucleocapsid negative (i.e., vaccinated/not infected) individuals than spike positive/nucleocapsid positive individuals (i.e., vaccinated/infected), however these differences were not statistically significant (p = 0.2). Spike antibody quantities were not statistically significantly associated with age, sex, the vaccine product received, or the number of doses received (highly colinear with time since last vaccine dose).

Among antibody positive individuals in the follow-up survey, antibody neutralization was high for all variants studied. Almost all (99%) participants had neutralizing antibodies to wild type (n = 593/599), alpha (n = 597/599), beta (n = 591/599), gamma (n = 594/599), and delta (n = 594/599) variants. Slightly fewer had neutralizing antibodies to omicron (n = 584/599; 97%). The levels of neutralizing antibodies were also very high, with at least 94% of samples yielding greater than 95% neutralization for each variant. Neutralizing levels were higher among individuals with evidence of natural infection (i.e. nucleocapsid positive/spike positive) compared to those who did not (i.e., nucleocapsid negative/spike positive; Table 4). These differences were most extreme for the omicron variant, such that the level of omicron neutralization was on average 14.8% higher among individuals with evidence of natural infection compared to those without. Neutralizing levels to omicron were also 3.8% higher among individuals with natural infection detected in this study compared to those with natural infection detected at the first survey (i.e., using 2020 sera).

**Table 4:** Levels of neutralizing antibody by variant, natural infection, and time point

SARS-CoV-2 Variant	Average percent neutralization among individuals with evidence of natural infection (n = 186) Mean (SD)	Average percent neutralization among individuals without evidence of natural infection (n = 413) Mean (SD)	Average percent neutralization among individuals with evidence of natural infection using 2020 sera (n = 100) Mean (SD)	Difference (%) in neutralization percentage between individuals with evidence of natural infection versus those without (95% CI) Mean (SD)	Difference (%) in neutralization percentage between individuals with evidence of natural infection between this study and 2020 sera (95% CI)
Wild type	98.3 (7.62)	96.5 (13.1)	90.1 (20.4)	1.7 (0.06, 3.4)	8.2 (4.1, 12.3)
Alpha	99.6 (1.79)	97.5 (11.9)	91.5 (18.6)	2.1 (0.9, 3.3)	8.1 (4.5, 11.8)
Beta	98.8 (5.34)	96.0 (15.9)	86.9 (27.2)	2.9 (1.2, 4.6)	11.9 (6.6, 17.3)
Gamma	98.5 (6.74)	96.0 (14.4)	90.0 (20.0)	2.5 (0.8, 4.2)	8.6 (4.5, 12.6)
Delta	99.3 (3.16)	96.8 (14.1)	87.3 (26.8)	2.6 (1.1, 4.0)	12.0 (6.8, 17.3)
Omicron	97.5 (7.31)	93.8 (18.5)	82.8 (31.1)	3.8 (1.7, 5.9)	14.8 (8.6, 20.9)

Household children were invited to participate, and 279 such children aged < 18 years were identified by their parents/guardians for possible enrollment into the study. 62 (22%) of these children completed the study, while 103 (37%) were unreachable, 73 (26%) declined participation, and 41 (15%) completed the survey but not the blood draw. Most pediatric participants (n = 41, 66.1%) were from Northwest Virginia and demographic characteristics largely matched the adult participants (Table S5).

Approximately half of pediatric participants (n = 35, 56.5%) were SARS-CoV-2 IgG nucleocapsid seropositive, and the majority (n = 54, 85.5%) were also SARS-CoV-2 IgG spike seropositive (Table S6). All vaccinated children were spike seropositive (n = 28/28). The majority of unvaccinated children were also spike seropositive (n = 22/30) with most of these being nucleocapsid seropositive indicating natural infection (n = 20/22). As with adults, vaccinated spike seropositive children had higher antibody quantities than unvaccinated spike seropositive children (88.5% of vaccinated children had spike quantities > 2500 U/ml compared to 10% of unvaccinated children).

Pediatric participants frequently reported at least one COVID-19-like illness (n = 45, 72.6%). Slightly more than half (n = 23) were tested for COVID-19 because of at least one of these illnesses, and 12 (19.4%) reported a positive result from this test (Table S7). All but one child who reported a positive test result were nucleocapsid seropositive. Being unvaccinated (n = 31 compared to n = 28 vaccinated) was associated with a 33% increased risk of being nucleocapsid positive (95% CI: -14%, 118%) among the pediatric participants. Nucleocapsid positivity in the parent was highly predictive of nucleocapsid positivity in the child: of 31 children whose parents were nucleocapsid seropositive, 27 were also nucleocapsid positive (87.1%), while among 30 children whose household adult were seronegative, only 8 were positive (26.7%; OR: 18.6, 95% CI: 4.9, 69.9). All children with neutralizing antibody testing (n = 41) were antibody positive for all variants studied, with average neutralizing antibody levels > 98% for all variants. There was no difference in neutralizing antibody levels between children who had evidence of natural infection versus those who did not.

## Discussion

In this longitudinal, demographically-representative, state-wide COVID-19 seroepidemiology study we noted a particularly highly vaccinated and boosted population and a low infection rate. Our rate of 93.4% of adults receiving at least one dose is slightly higher than the overall state rate of 92.2%, however more striking was that 77.0% of our study population had been boosted versus the state rate of 52.7% [6]. We believe this high rate was contributed by the health-seeking behavior of the study population, which consisted of individuals presenting for outpatient health care in 2020 and was further selected by being interested in participating in this follow-up study. Additionally, our study population consisted of a large number of elderly individuals, who are also a highly vaccinated demographic in our state.

The low natural infection rate of ~ 28.6% was also remarkable. The most recent data from Virginia from CDC surveys showed approximately 45-64% nucleocapsid seroprevalence as of 2022 [1]. There are several potential reasons for this comparatively low rate. First the high vaccination rate in our cohort may have yielded subsequent protection from infection. There was also some evidence that our study population exhibited behavioral modifications to decrease COVID-19 exposure, notably high N95 mask wearing rates and low rates of frequenting indoor bars. Moreover, our 28.6% rate is likely an underestimate for the true rate of prior COVID-19 infections across the state, since it is known that not 100% of infected individuals seroconvert, particularly after mild or asymptomatic infections [7–10]. It appears to be a particular underestimate for the Southwest region, which had the lowest seroprevalence in our study; however, total confirmed COVID-19 case counts per population are generally similar across the state (~ 25,000 per 100,000) and even slightly higher in the Southwest (~ 29,000/100,000) [11]. On the other hand it is notable that our population reported a 2.3% hospitalization rate for COVID-19-like illness. This is actually quite high, as state data has reported about 126,000 hospitalizations, which translates to approximately a 1.5% hospitalization rate for the state population [12]. Interestingly, children participants in this study had a higher rate of prior infection (56.5%) than adults. Whether this represents lower or later vaccine uptake or higher exposure risks will require further study.

Risk factors for infection were mostly expected. As with our first study and other studies, we noted lower rates in older populations and higher rates in Hispanics and Blacks and in those without health insurance. In the first study we noted higher rates in multi-family homes, and in this study we noted higher rates in households with higher numbers of children. These data along with the correlation between infection in parents/guardian and their children, suggest in-home exposures are highly important [1, 3].

Our spike antibody measurements were quantitative. While there is no specific antibody test or threshold that determines an individual’s risk of infection, a number of prior studies have shown that higher antibody quantities are associated with decreased risk of subsequent symptomatic COVID-19 infection [13]. Clearly other immune mechanisms besides serum IgG are important in preventing and limiting COVID19 infections, such as mucosal antibodies and T cells. Acknowledging this caveat, we noted that vaccinated/uninfected individuals had higher spike antibody quantities than vaccinated/infected individuals. This trend has been observed previously and clearly indicates the effectiveness of current vaccines to elicit strong antibody responses [14]. We also noted that spike positive/nucleocapsid positive individuals had notably higher neutralizing antibody responses to omicron than spike positive/nucleocapsid negative individuals. Since our population was > 93% vaccinated, these two populations reflect hybrid immunity (vaccinated/infected) versus vaccine-only immunity, respectively. This adds to a growing body of literature that shows the enhanced protection conferred by hybrid immunity [15–17] and underscores the importance of continuing vaccination strategies, particularly for the large vaccinated/uninfected population, since we and others have found that spike antibody responses clearly wane with time.

There were limitations to this study. First the portion of eligible individuals that chose to enroll and complete the study was low. Fortunately we were able to compare the demographics of the respondents with the first survey to know the bias and directionality of this subset. Additionally, our study design was that participants needed to undergo venipuncture, which may have favored individuals living near health care facilities, those that had greater means of transportation, or those that had high COVID-19 interests. That said we still systematically captured a large number of several hundred individuals from across a large geographic area. Further, while we corrected for imperfect sensitivity and specificity of the diagnostic testing in the seroprevalence estimates, the analysis of risk factors for seropositivity may have been biased by outcome misclassification. We expect this bias to be minimal given the excellent test characteristics of the assay used.

## Conclusions

In summary, we found a low natural infection rate in this highly vaccinated cohort from across Virginia. There are clearly large sectors of the population that have remained uninfected through July 2022, even after the peak of infections with the highly-transmissible omicron variant. Neutralizing antibodies are slightly lower in the vaccinated cohort versus those with hybrid immunity. Therefore for the large vaccinated/uninfected population, the durability of vaccine-induced antibodies, the importance of boosting, and potential susceptibility to new spike variants remain important to monitor.

### Electronic supplementary material

Below is the link to the electronic supplementary material.

Supplementary Material 1

## Data Availability

De-identified datasets used and/or analyzed during the current study are available from the corresponding author on reasonable request.
