# Supplementary material for: Risk factors and titers of COVID-19 infection in a longitudinal statewide seroepidemiology cohort
Source: BMC Infect Dis. 2023 Oct 11;23:676. doi: 10.1186/s12879-023-08670-6 (PMC10565985; doi:10.1186/s12879-023-08670-6)
Supplement: Supplementary file 1 — Supplementary Material 1 [file 12879_2023_8670_MOESM1_ESM.docx]

**Table S1. Characteristics of study population.**

|  | **Central**  **(N=90)** | **East**  **(N=206)** | **North**  **(N=81)** | **Northwest**  **(N=299)** | **Southwest**  **(N=108)** | **Overall**  **(N=784)** | **First survey data for comparison (N=4675)** |
| --- | --- | --- | --- | --- | --- | --- | --- |
| **Age** |  |  |  |  |  |  |  |
| 18-29 | 1 (1.1%) | 8 (3.9%) | 8 (9.9%) | 42 (14.0%) | 7 (6.5%) | 66 (8.4%) | 735 (15.7%) |
| 30-39 | 17 (18.9%) | 41 (19.9%) | 14 (17.3%) | 64 (21.4%) | 9 (8.3%) | 145 (18.5%) | 950 (20.3%) |
| 40-49 | 7 (7.8%) | 23 (11.2%) | 15 (18.5%) | 38 (12.7%) | 13 (12.0%) | 96 (12.2%) | 671 (14.4%) |
| 50-59 | 28 (31.1%) | 52 (25.2%) | 22 (27.2%) | 63 (21.1%) | 20 (18.5%) | 185 (23.6%) | 933 (20.0%) |
| 60-69 | 24 (26.7%) | 46 (22.3%) | 12 (14.8%) | 58 (19.4%) | 35 (32.4%) | 175 (22.3%) | 781 (16.7%) |
| 70-79 | 10 (11.1%) | 31 (15.0%) | 9 (11.1%) | 29 (9.7%) | 21 (19.4%) | 100 (12.8%) | 482 (10.3%) |
| 80+ | 3 (3.3%) | 5 (2.4%) | 1 (1.2%) | 5 (1.7%) | 3 (2.8%) | 17 (2.2%) | 123 (2.6%) |
| **Gender** |  |  |  |  |  |  |  |
| Female | 63 (70.0%) | 141 (68.4%) | 54 (66.7%) | 200 (66.9%) | 70 (64.8%) | 528 (67.3%) | 3119 (66.7%) |
| Male | 27 (30.0%) | 65 (31.6%) | 27 (33.3%) | 99 (33.1%) | 38 (35.2%) | 256 (32.7%) |  |
| **Race** |  |  |  |  |  |  |  |
| White | 58 (64.4%) | 150 (72.8%) | 55 (67.9%) | 259 (86.6%) | 94 (87.0%) | 616 (78.6%) | 3098 (66.3%) |
| African American | 23 (25.6%) | 41 (19.9%) | 7 (8.6%) | 18 (6.0%) | 9 (8.3%) | 98 (12.5%) | 942 (20.1%) |
| Asian | 2 (2.2%) | 4 (1.9%) | 13 (16.0%) | 7 (2.3%) | 0 (0.0%) | 26 (3.3%) | 281 (6.0%) |
| Other race | 0 (0.0%) | 0 (0.0%) | 0 (0.0%) | 0 (0.0%) | 0 (0.0%) | 0 (0.0%) | 278 (5.9%) |
| Two or more races | 0 (0.0%) | 0 (0.0%) | 0 (0.0%) | 0 (0.0%) | 0 (0.0%) | 0 (0.0%) | 76 (1.6%) |
| Missing | 7 (7.8%) | 11 (5.3%) | 6 (7.4%) | 15 (5.0%) | 5 (4.6%) | 44 (5.6%) |  |
| **Ethnicity** |  |  |  |  |  |  |  |
| Hispanic | 5 (5.6%) | 8 (3.9%) | 5 (6.2%) | 15 (5.0%) | 2 (1.9%) | 35 (4.5%) | 396 (8.5%) |
| non-Hispanic | 85 (94.4%) | 198 (96.1%) | 76 (93.8%) | 284 (95.0%) | 106 (98.1%) | 749 (95.5%) | 4279 (91.5%) |
| **Primary health insurance today** | | |  |  |  |  |  |
| Medicaid ^a^ | 5 (5.6%) | 10 (4.9%) | 2 (2.5%) | 34 (11.4%) | 16 (14.8%) | 67 (8.5%) | 530 (11.3%) |
| Medicare | 28 (31.1%) | 60 (29.1%) | 13 (16.0%) | 50 (16.7%) | 48 (44.4%) | 199 (25.4%) | 886 (19.0%) |
| Private (employer or individual) | 56 (62.2%) | 117 (56.8%) | 62 (76.5%) | 194 (64.9%) | 42 (38.9%) | 471 (60.1%) | 2857 (61.1%) |
| None or uninsured | 0 (0.0%) | 3 (1.5%) | 0 (0.0%) | 12 (4.0%) | 0 (0.0%) | 15 (1.9%) | 152 (3.3%) |
| Military ^b^ | 0 (0.0%) | 12 (5.8%) | 3 (3.7%) | 7 (2.3%) | 1 (0.9%) | 23 (2.9%) | 174 (3.7%) |
| Other | 1 (1.1%) | 3 (1.5%) | 1 (1.2%) | 2 (0.7%) | 1 (0.9%) | 8 (1.0%) |  |
| Don't know | 0 (0.0%) | 1 (0.5%) | 0 (0.0%) | 0 (0.0%) | 0 (0.0%) | 1 (0.1%) |  |
| **High risk health condition** ^c^ | | |  |  |  |  |  |
| Yes | 40 (44.4%) | 96 (46.6%) | 19 (23.5%) | 93 (31.1%) | 52 (48.1%) | 300 (38.3%) | 1854 (39.7%) |
| No | 50 (55.6%) | 109 (52.9%) | 62 (76.5%) | 204 (68.2%) | 55 (50.9%) | 480 (61.2%) | 2786 (59.6%) |
| Missing | 0 (0.0%) | 1 (0.5%) | 0 (0.0%) | 2 (0.7%) | 1 (0.9%) | 4 (0.5%) |  |
| **Type of dwelling** | |  |  |  |  |  |  |
| Single family | 79 (87.8%) | 166 (80.6%) | 52 (64.2%) | 248 (82.9%) | 90 (83.3%) | 635 (81.0%) | 3489 (74.6%) |
| Multi-family / Apartment / Condo building | 10 (11.1%) | 38 (18.4%) | 27 (33.3%) | 46 (15.4%) | 18 (16.7%) | 139 (17.7%) | 1144 (24.5%) |
| Long-term care facility or other congregate setting | 1 (1.1%) | 1 (0.5%) | 0 (0.0%) | 0 (0.0%) | 0 (0.0%) | 2 (0.3%) | 17 (0.4%) |
| Other | 0 (0.0%) | 1 (0.5%) | 2 (2.5%) | 5 (1.7%) | 0 (0.0%) | 8 (1.0%) |  |
| **Number of adults in household** | | |  |  |  |  |  |
| Mean (SD) | 2.2 (± 0.91) | 2.2 (± 0.94) | 2.1 (± 1.0) | 2.0 (± 0.92) | 2.0 (± 0.80) | 2.1 (± 0.92) | 2.2 (± 1.3) |
| **Number of children in household** | | | |  |  |  |  |
| Mean (SD) | 0.48 (± 1.0) | 0.37 (± 0.80) | 0.44 (± 0.82) | 0.46 (± 0.92) | 0.43 (± 0.89) | 0.43 (± 0.88) | 0.61 (± 1.0) |
| **Worked outside the home since June-August 2022** | | | | |  |  |  |
| Not at all | 43 (47.8%) | 79 (38.3%) | 36 (44.4%) | 120 (40.1%) | 65 (60.2%) | 343 (43.8%) |  |
| Up to 20 hours per week | 11 (12.2%) | 15 (7.3%) | 8 (9.9%) | 44 (14.7%) | 10 (9.3%) | 88 (11.2%) |  |
| More than 20 hours per week | 36 (40.0%) | 94 (45.6%) | 37 (45.7%) | 134 (44.8%) | 33 (30.6%) | 334 (42.6%) |  |
| Unknown | 0 (0.0%) | 18 (8.7%) | 0 (0.0%) | 1 (0.3%) | 0 (0.0%) | 19 (2.4%) |  |
| **Close contact with COVID+ individual** | | | |  |  |  |  |
| Yes | 34 (37.8%) | 86 (41.7%) | 43 (53.1%) | 135 (45.2%) | 44 (40.7%) | 342 (43.6%) | 367 (7.9%) |
| No | 49 (54.4%) | 74 (35.9%) | 29 (35.8%) | 145 (48.5%) | 56 (51.9%) | 353 (45.0%) |  |
| Don’t know | 7 (7.8%) | 46 (22.3%) | 9 (11.1%) | 19 (6.4%) | 8 (7.4%) | 89 (11.4%) |  |
| **Asked to quarantine for contact with COVID+ individual** | | | | | |  |  |
| Yes | 9 (10.0%) | 44 (21.4%) | 12 (14.8%) | 45 (15.1%) | 14 (13.0%) | 124 (15.8%) |  |
| No | 81 (90.0%) | 147 (71.4%) | 69 (85.2%) | 251 (83.9%) | 94 (87.0%) | 642 (81.9%) |  |
| Don’t know | 0 (0%) | 15 (7.3%) | 0 (0%) | 3 (1.0%) | 0 (0%) | 18 (2.3%) |  |
| **Frequency of indoor dining** | | |  |  |  |  |  |
| Never | 16 (17.8%) | 20 (9.7%) | 14 (17.3%) | 81 (27.1%) | 20 (18.5%) | 151 (19.3%) |  |
| About once a month | 34 (37.8%) | 87 (42.2%) | 37 (45.7%) | 136 (45.5%) | 39 (36.1%) | 333 (42.5%) |  |
| About once a week | 27 (30.0%) | 54 (26.2%) | 22 (27.2%) | 59 (19.7%) | 27 (25.0%) | 189 (24.1%) |  |
| A few times a week | 12 (13.3%) | 20 (9.7%) | 8 (9.9%) | 20 (6.7%) | 21 (19.4%) | 81 (10.3%) |  |
| Nearly every day | 0 (0.0%) | 4 (1.9%) | 0 (0.0%) | 2 (0.7%) | 1 (0.9%) | 7 (0.9%) |  |
| Unknown | 1 (1.1%) | 21 (10.2%) | 0 (0.0%) | 1 (0.3%) | 0 (0.0%) | 23 (2.9%) |  |
| **Frequency of visiting indoor bar** | | |  |  |  |  |  |
| Never | 67 (74.4%) | 147 (71.4%) | 51 (63.0%) | 230 (76.9%) | 87 (80.6%) | 582 (74.2%) |  |
| About once a month | 17 (18.9%) | 21 (10.2%) | 24 (29.6%) | 42 (14.0%) | 16 (14.8%) | 120 (15.3%) |  |
| About once a week | 5 (5.6%) | 12 (5.8%) | 3 (3.7%) | 18 (6.0%) | 4 (3.7%) | 42 (5.4%) |  |
| A few times a week | 1 (1.1%) | 4 (1.9%) | 3 (3.7%) | 3 (1.0%) | 1 (0.9%) | 12 (1.5%) |  |
| Nearly every day | 0 (0.0%) | 0 (0.0%) | 0 (0.0%) | 2 (0.7%) | 0 (0.0%) | 2 (0.3%) |  |
| Unknown | 0 (0.0%) | 22 (10.7%) | 0 (0.0%) | 4 (1.3%) | 0 (0.0%) | 26 (3.3%) |  |
| **Time indoors in public without a mask** | | | |  |  |  |  |
| Never | 28 (31.1%) | 67 (32.5%) | 41 (50.6%) | 157 (52.5%) | 29 (26.9%) | 322 (41.1%) |  |
| About once a month | 13 (14.4%) | 27 (13.1%) | 17 (21.0%) | 38 (12.7%) | 18 (16.7%) | 113 (14.4%) |  |
| About once a week | 21 (23.3%) | 29 (14.1%) | 7 (8.6%) | 40 (13.4%) | 14 (13.0%) | 111 (14.2%) |  |
| A few times a week | 20 (22.2%) | 34 (16.5%) | 11 (13.6%) | 40 (13.4%) | 33 (30.6%) | 138 (17.6%) |  |
| Nearly every day | 7 (7.8%) | 28 (13.6%) | 4 (4.9%) | 23 (7.7%) | 13 (12.0%) | 75 (9.6%) |  |
| Unknown | 1 (1.1%) | 21 (10.2%) | 1 (1.2%) | 1 (0.3%) | 1 (0.9%) | 25 (3.2%) |  |
| **Time indoors in public with others not wearing masks** | | | | |  |  |  |
| All of the time | 49 (54.4%) | 78 (37.9%) | 6 (7.4%) | 102 (34.1%) | 51 (47.2%) | 286 (36.5%) |  |
| Most of the time (>50%) | 26 (28.9%) | 63 (30.6%) | 26 (32.1%) | 84 (28.1%) | 48 (44.4%) | 247 (31.5%) |  |
| Some of the time (<50%) | 14 (15.6%) | 39 (18.9%) | 44 (54.3%) | 100 (33.4%) | 8 (7.4%) | 205 (26.1%) |  |
| None of the time | 1 (1.1%) | 3 (1.5%) | 5 (6.2%) | 11 (3.7%) | 1 (0.9%) | 21 (2.7%) |  |
| Unknown | 0 (0.0%) | 23 (11.2%) | 0 (0.0%) | 2 (0.7%) | 0 (0.0%) | 25 (3.2%) |  |
| **Received COVID-19 vaccine** | | |  |  |  |  |  |
| Yes | 89 (98.9%) | 184 (89.3%) | 78 (96.3%) | 279 (93.3%) | 102 (94.4%) | 732 (93.4%) |  |
| No | 1 (1.1%) | 10 (4.9%) | 3 (3.7%) | 19 (6.4%) | 6 (5.6%) | 39 (5.0%) |  |
| Don't know | 0 (0.0%) | 12 (5.8%) | 0 (0.0%) | 1 (0.3%) | 0 (0.0%) | 13 (1.7%) |  |
| **Number of COVID-19 vaccine doses received** | | | | |  |  |  |
| 1 | 1 (1.1%) | 4 (1.9%) | 0 (0.0%) | 7 (2.3%) | 4 (3.7%) | 16 (2.0%) |  |
| 2 | 11 (12.2%) | 39 (18.9%) | 11 (13.6%) | 57 (19.1%) | 22 (20.4%) | 140 (17.9%) |  |
| 3 | 58 (64.4%) | 126 (61.2%) | 66 (81.5%) | 210 (70.2%) | 63 (58.3%) | 523 (66.7%) |  |
| 4 | 19 (21.1%) | 13 (6.3%) | 0 (0.0%) | 5 (1.7%) | 13 (12.0%) | 50 (6.4%) |  |
| Unknown | 0 (0.0%) | 2 (1.0%) | 1 (1.2%) | 0 (0.0%) | 0 (0.0%) | 3 (0.4%) |  |
| N/A | 1 (1.1%) | 22 (10.7%) | 3 (3.7%) | 20 (6.7%) | 6 (5.6%) | 52 (6.6%) |  |
| **COVID-19 vaccine received (dose 1)** | | | |  |  |  |  |
| Pfizer | 53 (58.9%) | 101 (49.0%) | 53 (65.4%) | 156 (52.2%) | 54 (50.0%) | 417 (53.2%) |  |
| Moderna | 32 (35.6%) | 73 (35.4%) | 22 (27.2%) | 100 (33.4%) | 36 (33.3%) | 263 (33.5%) |  |
| Johnson and Johnson | 2 (2.2%) | 8 (3.9%) | 3 (3.7%) | 21 (7.0%) | 12 (11.1%) | 46 (5.9%) |  |
| Other | 1 (1.1%) | 1 (0.5%) | 0 (0.0%) | 0 (0.0%) | 0 (0.0%) | 2 (0.3%) |  |
| Unknown | 1 (1.1%) | 1 (0.5%) | 0 (0.0%) | 2 (0.7%) | 0 (0.0%) | 4 (0.5%) |  |
| N/A | 1 (1.1%) | 22 (10.7%) | 3 (3.7%) | 20 (6.7%) | 6 (5.6%) | 52 (6.6%) |  |
| **Time outdoors in public with a mask** | | | |  |  |  |  |
| All of the time | 24 (26.7%) | 48 (23.3%) | 30 (37.0%) | 97 (32.4%) | 19 (17.6%) | 218 (27.8%) |  |
| Most of the time (>50%) | 13 (14.4%) | 34 (16.5%) | 25 (30.9%) | 61 (20.4%) | 18 (16.7%) | 151 (19.3%) |  |
| Some of the time (<50%) | 23 (25.6%) | 41 (19.9%) | 13 (16.0%) | 71 (23.7%) | 29 (26.9%) | 177 (22.6%) |  |
| None of the time | 30 (33.3%) | 59 (28.6%) | 13 (16.0%) | 67 (22.4%) | 39 (36.1%) | 208 (26.5%) |  |
| Unknown | 0 (0.0%) | 24 (11.7%) | 0 (0.0%) | 3 (1.0%) | 3 (2.8%) | 30 (3.8%) |  |
| **Type of mask worn most often** | | |  |  |  |  |  |
| Surgical mask | 42 (46.7%) | 67 (32.5%) | 33 (40.7%) | 141 (47.2%) | 38 (35.2%) | 321 (40.9%) |  |
| Cloth mask | 11 (12.2%) | 63 (30.6%) | 26 (32.1%) | 73 (24.4%) | 37 (34.3%) | 210 (26.8%) |  |
| Single layer mask/neck gaiter | 2 (2.2%) | 4 (1.9%) | 2 (2.5%) | 2 (0.7%) | 2 (1.9%) | 12 (1.5%) |  |
| N95/KN95 | 32 (35.6%) | 35 (17.0%) | 17 (21.0%) | 67 (22.4%) | 26 (24.1%) | 177 (22.6%) |  |
| Mask with exhalation valve | 0 (0.0%) | 0 (0.0%) | 1 (1.2%) | 0 (0.0%) | 1 (0.9%) | 2 (0.3%) |  |
| Missing | 3 (3.3%) | 37 (18.0%) | 2 (2.5%) | 16 (5.4%) | 4 (3.7%) | 62 (7.9%) |  |
| **Frequency mask completely covers nose and mouth** | | | | |  |  |  |
| All of the time | 81 (90.0%) | 164 (79.6%) | 66 (81.5%) | 278 (93.0%) | 96 (88.9%) | 685 (87.4%) |  |
| Most of the time (>50%) | 6 (6.7%) | 15 (7.3%) | 11 (13.6%) | 14 (4.7%) | 8 (7.4%) | 54 (6.9%) |  |
| Some of the time (<50%) | 2 (2.2%) | 7 (3.4%) | 4 (4.9%) | 2 (0.7%) | 1 (0.9%) | 16 (2.0%) |  |
| None of the time | 1 (1.1%) | 2 (1.0%) | 0 (0.0%) | 3 (1.0%) | 0 (0.0%) | 6 (0.8%) |  |
| Unknown | 0 (0.0%) | 18 (8.7%) | 0 (0.0%) | 2 (0.7%) | 3 (2.8%) | 23 (2.9%) |  |

^a^ Medicaid includes FAMIS, Virginia’s health insurance program for children

^b^ includes Tricare or Veterans Administration

^c^ diabetes, lung disease (including moderate to severe asthma), a severe heart condition, kidney disease, liver disease, or immunocompromise

**Table S2. Seroprevalence and titer levels among adult participants.**

|  | **Central**  **(N=90)** | **East**  **(N=206)** | **North**  **(N=81)** | **Northwest**  **(N=299)** | **Southwest**  **(N=108)** | **Overall**  **(N=784)** |
| --- | --- | --- | --- | --- | --- | --- |
| **Nucleocapsid result** | | |  |  |  |  |
| Negative | 59 (65.6%) | 127 (61.7%) | 65 (80.2%) | 214 (71.6%) | 93 (86.1%) | 558 (71.2%) |
| Positive | 31 (34.4%) | 79 (38.3%) | 16 (19.8%) | 83 (27.8%) | 15 (13.9%) | 224 (28.6%) |
| Missing | 0 (0%) | 0 (0%) | 0 (0%) | 2 (0.7%) | 0 (0%) | 2 (0.3%) |
| **Spike result** | |  |  |  |  |  |
| Negative | 5 (5.6%) | 4 (1.9%) | 2 (2.5%) | 9 (3.0%) | 2 (1.9%) | 22 (2.8%) |
| Positive | 85 (94.4%) | 201 (97.6%) | 79 (97.5%) | 290 (97.0%) | 105 (97.2%) | 760 (96.9%) |
| Missing | 0 (0%) | 1 (0.5%) | 0 (0%) | 0 (0%) | 1 (0.9%) | 2 (0.3%) |
| **Spike quantity** | |  |  |  |  |  |
| Negative (< 0.80 U/ml) | 5 (5.6%) | 5 (2.4%) | 2 (2.5%) | 9 (3.0%) | 3 (2.8%) | 24 (3.1%) |
| 0.8 to 2500 U/ml | 18 (20.0%) | 58 (28.2%) | 14 (17.3%) | 60 (20.1%) | 33 (30.6%) | 183 (23.3%) |
| >2500 U/ml | 67 (74.4%) | 140 (68.0%) | 62 (76.5%) | 163 (54.5%) | 72 (66.7%) | 504 (64.3%) |
| Missing | 0 (0%) | 3 (1.5%) | 3 (3.7%) | 67 (22.4%) | 0 (0%) | 73 (9.3%) |

**Table S3. Report of COVID-like illness and test result history in the study population.**

|  | **Central**  **(N=90)** | **East**  **(N=206)** | **North**  **(N=81)** | **Northwest**  **(N=299)** | **Southwest**  **(N=108)** | **Overall**  **(N=784)** |
| --- | --- | --- | --- | --- | --- | --- |
| **Self-report COVID-like illness since prior survey** | | | | | |  |
| Yes | 38 (42.2%) | 93 (45.1%) | 23 (28.4%) | 134 (44.8%) | 41 (38.0%) | 329 (42.0%) |
| No | 52 (57.8%) | 100 (48.5%) | 56 (69.1%) | 162 (54.2%) | 66 (61.1%) | 436 (55.6%) |
| Don't know or can't remember | 0 (0.0%) | 1 (0.5%) | 2 (2.5%) | 2 (0.7%) | 1 (0.9%) | 6 (0.8%) |
| Missing | 0 (0%) | 12 (5.8%) | 0 (0%) | 1 (0.3%) | 0 (0%) | 13 (1.7%) |
| **Called or went to a doctor, clinic, or emergency room because of these illnesses** | | | | | | |
| Yes | 22 (24.4%) | 54 (26.2%) | 12 (14.8%) | 86 (28.8%) | 19 (17.6%) | 193 (24.6%) |
| No | 16 (17.8%) | 38 (18.4%) | 11 (13.6%) | 48 (16.1%) | 22 (20.4%) | 135 (17.2%) |
| Don't know or can't remember | 0 (0.0%) | 13 (6.3%) | 0 (0.0%) | 1 (0.3%) | 0 (0.0%) | 14 (1.8%) |
| NA | 52 (57.8%) | 101 (49.0%) | 58 (71.6%) | 164 (54.8%) | 67 (62.0%) | 442 (56.4%) |
| **Hospitalized for any of these illnesses** | | | | |  |  |
| Yes | 1 (1.1%) | 7 (3.4%) | 1 (1.2%) | 7 (2.3%) | 2 (1.9%) | 18 (2.3%) |
| No | 21 (23.3%) | 47 (22.8%) | 11 (13.6%) | 79 (26.4%) | 17 (15.7%) | 175 (22.3%) |
| Don't know or can't remember | 0 (0.0%) | 0 (0.0%) | 0 (0.0%) | 0 (0.0%) | 0 (0.0%) | 0 (0.0%) |
| NA | 68 (75.6%) | 152 (73.8%) | 69 (85.2%) | 213 (71.2%) | 89 (82.4%) | 591 (75.4%) |
| **Tested for COVID for any of these illnesses** | | | | |  |  |
| Yes | 32 (35.6%) | 62 (30.1%) | 14 (17.3%) | 93 (31.1%) | 28 (25.9%) | 229 (29.2%) |
| No | 6 (6.7%) | 10 (4.9%) | 1 (1.2%) | 6 (2.0%) | 7 (6.5%) | 30 (3.8%) |
| Unknown | 0 (0.0%) | 34 (16.5%) | 10 (12.3%) | 38 (12.7%) | 7 (6.5%) | 89 (11.4%) |
| Not applicable | 52 (57.8%) | 100 (48.5%) | 56 (69.1%) | 162 (54.2%) | 66 (61.1%) | 436 (55.6%) |
| **Test result for reported illness** | | | |  |  |  |
| Positive | 17 (18.9%) | 34 (16.5%) | 9 (11.1%) | 44 (14.7%) | 15 (13.9%) | 119 (15.2%) |
| Negative | 14 (15.6%) | 28 (13.6%) | 5 (6.2%) | 48 (16.1%) | 13 (12.0%) | 108 (13.8%) |
| Have not received result | 0 (0.0%) | 0 (0.0%) | 0 (0.0%) | 0 (0.0%) | 0 (0.0%) | 0 (0.0%) |
| Don't know | 1 (1.1%) | 0 (0.0%) | 0 (0.0%) | 1 (0.3%) | 0 (0.0%) | 2 (0.3%) |
| NA | 58 (64.4%) | 144 (69.9%) | 67 (82.7%) | 206 (68.9%) | 80 (74.1%) | 555 (70.8%) |
| **Number of times tested for COVID for any other reason** | | | | | | |
| No tests | 13 (14.4%) | 50 (24.3%) | 16 (19.8%) | 70 (23.4%) | 28 (25.9%) | 177 (22.6%) |
| 1-2 tests | 22 (24.4%) | 83 (40.3%) | 29 (35.8%) | 116 (38.8%) | 41 (38.0%) | 291 (37.1%) |
| 3-10 tests | 50 (55.6%) | 53 (25.7%) | 30 (37.0%) | 87 (29.1%) | 36 (33.3%) | 256 (32.7%) |
| >10 tests | 5 (5.6%) | 5 (2.4%) | 5 (6.2%) | 24 (8.0%) | 2 (1.9%) | 41 (5.2%) |
| Missing | 0 (0%) | 15 (7.3%) | 1 (1.2%) | 2 (0.7%) | 1 (0.9%) | 19 (2.4%) |
| **Positive COVID test for any other reason** | | | | |  |  |
| No | 79 (87.8%) | 86 (41.7%) | 19 (23.5%) | 74 (24.7%) | 71 (65.7%) | 329 (42.0%) |
| Question not asked | 9 (10.0%) | 119 (57.8%) | 62 (76.5%) | 222 (74.2%) | 32 (29.6%) | 444 (56.6%) |
| Yes | 2 (2.2%) | 1 (0.5%) | 0 (0.0%) | 3 (1.0%) | 5 (4.6%) | 11 (1.4%) |

**Table S4. SARS-CoV-2 spike seroprevalence across Virginia by geography and subgroup**

| **Region/subgroup** | **Number of participants** | **Number seropositive** | **Raw prevalence (%)** | **Adjusted^d^ Prevalence (95% CI)** | **Adjusted^d,e^ and corrected Prevalence (95% CI)** |
| --- | --- | --- | --- | --- | --- |
| Geographical Region |  |  |  |  |  |
| Central | 90 | 85 | 94.4 | 94.8 (89.6, 100.0) | 102.3 (96.6, 107.9) |
| East | 205 | 201 | 98 | 97.9 (95.8, 100.0) | 105.6 (103.4, 107.9) |
| North | 81 | 79 | 97.5 | 98.1 (95.1, 101.2) | 105.9 (102.6, 109.2) |
| Northwest | 299 | 290 | 97 | 97.3 (95.3, 99.2) | 104.9 (102.8, 107.0) |
| Southwest | 107 | 105 | 98.1 | 98.8 (96.7, 100.8) | 106.5 (104.3, 108.8) |
| Virginia (Overall) | 782 | 760 | 97.2 | 97.5 (96.1, 98.9) | 105.2 (103.6, 106.7) |
| Age (years) |  |  |  |  |  |
| 18-29 | 66 | 62 | 93.9 | 98.3 (96.2, 100.4) | 106.0 (103.8, 108.3) |
| 30-39 | 145 | 143 | 98.6 | 99.2 (98.1, 100.3) | 107.0 (105.8, 108.2) |
| 40-49 | 96 | 95 | 99 | 99.6 (98.7, 100.4) | 107.4 (106.5, 108.3) |
| 50-59 | 185 | 175 | 94.6 | 94.1 (89.8, 98.4) | 101.6 (96.9, 106.2) |
| 60-69 | 173 | 170 | 98.3 | 97.6 (94.3, 100.9) | 105.3 (101.7, 108.8) |
| 70-79 | 100 | 99 | 99 | 99.5 (98.4, 100.5) | 107.3 (106.2, 108.4) |
| ≥80 | 17 | 16 | 94.1 | 91.9 (76.7, 107.1) | 99.1 (82.7, 115.6) |
| Gender |  |  |  |  |  |
| Female | 527 | 509 | 96.6 | 96.9 (95.3, 98.5) | 104.6 (102.8, 106.3) |
| Male | 255 | 251 | 98.4 | 98.1 (95.7, 100.4) | 105.8 (103.2, 108.4) |
| Race |  |  |  |  |  |
| White | 616 | 596 | 96.8 | 96.7 (94.7, 98.7) | 104.3 (102.1, 106.4) |
| African American | 96 | 95 | 99 | 98.9 (96.8, 101.1) | 106.7 (104.4, 109.0) |
| Asian | 26 | 26 | 100 | 100.0 (100.0, 100.0) | 107.9 (107.7, 108.1) |
| Two or more races | 12 | 11 | 91.7 | 95.9 (87.8, 104.1) | 103.5 (94.7, 112.3) |
| Other race | 32 | 32 | 100 | 100.0 (100.0, 100.0) | 107.9 (107.7, 108.1) |
| Ethnicity |  |  |  |  |  |
| Hispanic | 35 | 34 | 97.1 | 93.1 (79.8, 106.4) | 100.4 (86.0, 114.7) |
| non-Hispanic | 747 | 726 | 97.2 | 97.7 (96.4, 99.0) | 105.4 (104.0, 106.8) |
| Health-insurance at time of survey^a^ |  |  |  |  |  |
| Medicaid^b^ | 75 | 70 | 93.3 | 96.4 (92.9, 99.8) | 104.0 (100.3, 107.6) |
| Medicare | 67 | 61 | 91 | 94.4 (89.4, 99.3) | 99.7 (91.6, 107.9) |
| Private (employer or individual) | 470 | 463 | 98.5 | 98.5 (97.1, 99.9) | 106.3 (104.8, 107.8) |
| Military, Tricare, or Veterans Administration | 23 | 22 | 95.7 | 93.6 (81.5, 105.8) | 101.0 (87.9, 114.1) |
| None or uninsured | 15 | 14 | 93.3 | 96.3 (89.0, 103.6) | 103.9 (96.0, 111.8) |
| High-Risk Health Condition^c^ |  |  |  |  |  |
| Yes | 299 | 288 | 96.3 | 96.9 (94.9, 99.0) | 104.6 (102.3, 106.8) |
| No | 479 | 468 | 97.7 | 97.7 (95.8, 99.6) | 105.4 (103.3, 107.4) |

^a^ Health insurance missing for 9 individuals

^b^ Medicaid includes FAMIS, Virginia’s health insurance program for children

^c^ diabetes, lung disease (including moderate to severe asthma), a severe heart condition, kidney disease, liver disease, or immunocompromised; missing for 4 individuals

^d^ Reweighted by region, age, and sex to match regional population estimates and

^e^ Corrected for imperfect sensitivity and specificity of diagnostic tests

**Table S5. Characteristics of pediatric participants.**

|  | Central  (N=2) | East  (N=4) | North  (N=5) | Northwest  (N=41) | Southwest  (N=10) | Overall  (N=62) |
| --- | --- | --- | --- | --- | --- | --- |
| **Age** |  |  |  |  |  |  |
| Mean (SD) | 10 (± 5.7) | 10 (± 6.0) | 13 (± 3.6) | 8.5 (± 6.2) | 9.5 (± 3.7) | 9.2 (± 5.7) |
| Missing | 0 (0%) | 0 (0%) | 0 (0%) | 1 (2.4%) | 0 (0%) | 1 (1.6%) |
| **Gender** |  |  |  |  |  |  |
| Male | 1 (50.0%) | 2 (50.0%) | 3 (60.0%) | 22 (53.7%) | 7 (70.0%) | 35 (56.5%) |
| Female | 1 (50.0%) | 2 (50.0%) | 2 (40.0%) | 18 (43.9%) | 3 (30.0%) | 26 (41.9%) |
| Missing | 0 (0%) | 0 (0%) | 0 (0%) | 1 (2.4%) | 0 (0%) | 1 (1.6%) |
| **Race/ethnicity** | |  |  |  |  |  |
| White/non-hispanic | 1 (50.0%) | 2 (50.0%) | 1 (20.0%) | 30 (73.2%) | 6 (60.0%) | 40 (64.5%) |
| African American | 1 (50.0%) | 1 (25.0%) | 0 (0.0%) | 6 (14.6%) | 1 (10.0%) | 9 (14.5%) |
| Asian | 0 (0.0%) | 0 (0.0%) | 3 (60.0%) | 0 (0.0%) | 0 (0.0%) | 3 (4.8%) |
| Hispanic white | 0 (0.0%) | 0 (0.0%) | 1 (20.0%) | 1 (2.4%) | 0 (0.0%) | 2 (3.2%) |
| Other race | 0 (0.0%) | 1 (25.0%) | 0 (0.0%) | 3 (7.3%) | 3 (30.0%) | 7 (11.3%) |
| Missing | 0 (0%) | 0 (0%) | 0 (0%) | 1 (2.4%) | 0 (0%) | 1 (1.6%) |
| **High risk health condition** | | |  |  |  |  |
| Yes | 0 (0.0%) | 1 (25.0%) | 1 (20.0%) | 3 (7.3%) | 1 (10.0%) | 6 (9.7%) |
| No | 2 (100.0%) | 3 (75.0%) | 4 (80.0%) | 37 (90.2%) | 9 (90.0%) | 55 (88.7%) |
| Missing | 0 (0%) | 0 (0%) | 0 (0%) | 1 (2.4%) | 0 (0%) | 1 (1.6%) |
| **Attends school** | |  |  |  |  |  |
| Yes | 2 (100.0%) | 3 (75.0%) | 5 (100.0%) | 29 (70.7%) | 10 (100.0%) | 49 (79.0%) |
| No | 0 (0.0%) | 1 (25.0%) | 0 (0.0%) | 11 (26.8%) | 0 (0.0%) | 12 (19.4%) |
| Missing | 0 (0%) | 0 (0%) | 0 (0%) | 1 (2.4%) | 0 (0%) | 1 (1.6%) |
| **Close contact with COVID+ individual** | | | | |  |  |
| Yes | 1 (50.0%) | 2 (50.0%) | 3 (60.0%) | 19 (46.3%) | 7 (70.0%) | 32 (51.6%) |
| No | 1 (50.0%) | 1 (25.0%) | 2 (40.0%) | 15 (36.6%) | 1 (10.0%) | 20 (32.3%) |
| Don't know | 0 (0.0%) | 1 (25.0%) | 0 (0.0%) | 7 (17.1%) | 2 (20.0%) | 10 (16.1%) |
| **Asked to quarantine for contact with COVID+ individual** | | | | | | |
| Yes | 0 (0.0%) | 1 (25.0%) | 2 (40.0%) | 15 (36.6%) | 5 (50.0%) | 23 (37.1%) |
| No | 2 (100.0%) | 3 (75.0%) | 3 (60.0%) | 25 (61.0%) | 5 (50.0%) | 38 (61.3%) |
| Don't know | 0 (0.0%) | 0 (0.0%) | 0 (0.0%) | 1 (2.4%) | 0 (0.0%) | 1 (1.6%) |
| **Frequency of indoor dining** | | | |  |  |  |
| Never | 1 (50.0%) | 2 (50.0%) | 0 (0.0%) | 20 (48.8%) | 2 (20.0%) | 25 (40.3%) |
| About once a month | 0 (0.0%) | 1 (25.0%) | 2 (40.0%) | 15 (36.6%) | 6 (60.0%) | 24 (38.7%) |
| About once a week | 0 (0.0%) | 1 (25.0%) | 1 (20.0%) | 0 (0.0%) | 2 (20.0%) | 4 (6.5%) |
| A few times a week | 1 (50.0%) | 0 (0.0%) | 2 (40.0%) | 1 (2.4%) | 0 (0.0%) | 4 (6.5%) |
| Nearly every day | 0 (0.0%) | 0 (0.0%) | 0 (0.0%) | 4 (9.8%) | 0 (0.0%) | 4 (6.5%) |
| Unknown | 0 (0.0%) | 0 (0.0%) | 0 (0.0%) | 1 (2.4%) | 0 (0.0%) | 1 (1.6%) |
| **Received COVID-19 vaccine** | | | |  |  |  |
| Yes | 2 (100.0%) | 2 (50.0%) | 4 (80.0%) | 15 (36.6%) | 5 (50.0%) | 28 (45.2%) |
| No | 0 (0.0%) | 2 (50.0%) | 1 (20.0%) | 23 (56.1%) | 5 (50.0%) | 31 (50.0%) |
| Don't know | 0 (0.0%) | 0 (0.0%) | 0 (0.0%) | 3 (7.3%) | 0 (0.0%) | 3 (4.8%) |
| **Type of mask worn most often** | | | |  |  |  |
| Surgical mask | 1 (50.0%) | 0 (0.0%) | 4 (80.0%) | 19 (46.3%) | 4 (40.0%) | 28 (45.2%) |
| Cloth mask | 0 (0.0%) | 3 (75.0%) | 0 (0.0%) | 6 (14.6%) | 5 (50.0%) | 14 (22.6%) |
| Single layer mask/neck gaiter | 0 (0.0%) | 0 (0.0%) | 0 (0.0%) | 0 (0.0%) | 0 (0.0%) | 0 (0.0%) |
| N95/KN95 | 1 (50.0%) | 0 (0.0%) | 1 (20.0%) | 7 (17.1%) | 0 (0.0%) | 9 (14.5%) |
| Mask with exhalation valve | 0 (0.0%) | 0 (0.0%) | 0 (0.0%) | 0 (0.0%) | 0 (0.0%) | 0 (0.0%) |
| Other | 0 (0.0%) | 1 (25.0%) | 0 (0.0%) | 1 (2.4%) | 0 (0.0%) | 2 (3.2%) |
| Unsure | 0 (0.0%) | 0 (0.0%) | 0 (0.0%) | 8 (19.5%) | 1 (10.0%) | 9 (14.5%) |
| **Frequency mask completely covers nose and mouth** | | | | | |  |
| All of the time | 1 (50.0%) | 3 (75.0%) | 4 (80.0%) | 22 (53.7%) | 5 (50.0%) | 35 (56.5%) |
| Most of the time (>50%) | 0 (0.0%) | 0 (0.0%) | 1 (20.0%) | 5 (12.2%) | 3 (30.0%) | 9 (14.5%) |
| Some of the time (<50%) | 0 (0.0%) | 0 (0.0%) | 0 (0.0%) | 2 (4.9%) | 1 (10.0%) | 3 (4.8%) |
| None of the time | 1 (50.0%) | 1 (25.0%) | 0 (0.0%) | 7 (17.1%) | 1 (10.0%) | 10 (16.1%) |
| Unknown | 0 (0.0%) | 0 (0.0%) | 0 (0.0%) | 5 (12.2%) | 0 (0.0%) | 5 (8.1%) |

**Table S6. Seroprevalence and titer levels among pediatric participants.**

|  | **Central**  **(N=2)** | **East**  **(N=4)** | **North**  **(N=5)** | **Northwest**  **(N=41)** | **Southwest**  **(N=10)** | **Overall**  **(N=62)** | |
| --- | --- | --- | --- | --- | --- | --- | --- |
| **Nucleocapsid result** | | |  |  |  |  |  |
| Negative | 1 (50.0%) | 2 (50.0%) | 1 (20.0%) | 16 (39.0%) | 6 (60.0%) | 26 (41.9%) | |
| Positive | 1 (50.0%) | 2 (50.0%) | 4 (80.0%) | 24 (58.5%) | 4 (40.0%) | 35 (56.5%) | |
| Missing | 0 (0%) | 0 (0%) | 0 (0%) | 1 (2.4%) | 0 (0%) | 1 (1.6%) | |
| **Spike result** | |  |  |  |  |  |  |
| Negative | 0 (0.0%) | 1 (25.0%) | 1 (20.0%) | 4 (9.8%) | 2 (20.0%) | 8 (12.9%) | |
| Positive | 2 (100.0%) | 3 (75.0%) | 4 (80.0%) | 36 (87.8%) | 8 (80.0%) | 53 (85.5%) | |
| Missing | 0 (0%) | 0 (0%) | 0 (0%) | 1 (2.4%) | 0 (0%) | 1 (1.6%) | |
| **Spike quantity** | |  |  |  |  |  |  |
| Negative (< 0.80 U/ml) | 0 (0.0%) | 1 (25.0%) | 1 (20.0%) | 5 (12.2%) | 2 (20.0%) | 9 (14.5%) | |
| Positive 0.8 to 2500 U/ml | 1 (50.0%) | 1 (25.0%) | 0 (0.0%) | 17 (41.5%) | 3 (30.0%) | 22 (35.5%) | |
| Positive >2500 U/ml | 1 (50.0%) | 2 (50.0%) | 3 (60.0%) | 14 (34.1%) | 5 (50.0%) | 25 (40.3%) | |
| Missing | 0 (0%) | 0 (0%) | 1 (20.0%) | 5 (12.2%) | 0 (0%) | 6 (9.7%) | |
| **Nucleocapsid cut-off index** | | |  |  |  |  |  |
| Negative (<1.0) | 1 (50.0%) | 2 (50.0%) | 1 (20.0%) | 15 (36.6%) | 0 (0.0%) | 19 (30.6%) | |
| Positive (1.0-100) | 1 (50.0%) | 1 (25.0%) | 3 (60.0%) | 16 (39.0%) | 0 (0.0%) | 21 (33.9%) | |
| Positive >100 | 0 (0.0%) | 1 (25.0%) | 1 (20.0%) | 8 (19.5%) | 0 (0.0%) | 10 (16.1%) | |
| Missing | 0 (0%) | 0 (0%) | 0 (0%) | 2 (4.9%) | 10 (100%) | 12 (19.4%) | |

**Table S7. Report of COVID-like illness and test result history in the pediatric study population.**

|  | **Central**  **(N=2)** | **East**  **(N=4)** | **North**  **(N=5)** | **Northwest**  **(N=41)** | | **Southwest**  **(N=10)** | | **Overall**  **(N=62)** | |  |
| --- | --- | --- | --- | --- | --- | --- | --- | --- | --- | --- |
| **Self-report COVID-like illness since prior survey** | | | | | | | | |  | |
| Yes | 2 (100.0%) | 4 (100.0%) | 4 (80.0%) | 29 (70.7%) | | 6 (60.0%) | | 45 (72.6%) | |  |
| No | 0 (0.0%) | 0 (0.0%) | 1 (20.0%) | 11 (26.8%) | | 4 (40.0%) | | 16 (25.8%) | |  |
| Don't know or can't remember | 0 (0.0%) | 0 (0.0%) | 0 (0.0%) | 1 (2.4%) | | 0 (0.0%) | | 1 (1.6%) | |  |
| **Called or went to a doctor, clinic, or emergency room because of these illnesses** | | | | | | | | | | |
| Yes | 0 (0.0%) | 3 (75.0%) | 2 (40.0%) | 15 (36.6%) | | 4 (40.0%) | | 24 (38.7%) | |  |
| No | 2 (100.0%) | 1 (25.0%) | 2 (40.0%) | 14 (34.1%) | | 2 (20.0%) | | 21 (33.9%) | |  |
| NA | 0 (0.0%) | 0 (0.0%) | 1 (20.0%) | 12 (29.3%) | | 4 (40.0%) | | 17 (27.4%) | |  |
| **Hospitalized for any of these illnesses** | | | | | | |  | |  | |
| Yes | 0 (0.0%) | 0 (0.0%) | 0 (0.0%) | 0 (0.0%) | | 0 (0.0%) | | 0 (0.0%) | |  |
| No | 0 (0.0%) | 3 (75.0%) | 2 (40.0%) | 15 (36.6%) | | 4 (40.0%) | | 24 (38.7%) | |  |
| NA | 2 (100.0%) | 1 (25.0%) | 3 (60.0%) | 26 (63.4%) | | 6 (60.0%) | | 38 (61.3%) | |  |
| **Tested for COVID for any of these illnesses** | | | | | | |  | |  | |
| Yes | 0 (0.0%) | 3 (75.0%) | 2 (40.0%) | 14 (34.1%) | | 4 (40.0%) | | 23 (37.1%) | |  |
| No | 0 (0.0%) | 0 (0.0%) | 0 (0.0%) | 1 (2.4%) | | 0 (0.0%) | | 1 (1.6%) | |  |
| Unknown | 2 (100.0%) | 1 (25.0%) | 2 (40.0%) | 15 (36.6%) | | 2 (20.0%) | | 22 (35.5%) | |  |
| Not applicable | 0 (0.0%) | 0 (0.0%) | 1 (20.0%) | 11 (26.8%) | | 4 (40.0%) | | 16 (25.8%) | |  |
| **Test result for reported illness** | | | | |  | |  | |  | |
| Positive | 0 (0.0%) | 0 (0.0%) | 1 (20.0%) | 7 (17.1%) | | 4 (40.0%) | | 12 (19.4%) | |  |
| Negative | 0 (0.0%) | 3 (75.0%) | 1 (20.0%) | 7 (17.1%) | | 0 (0.0%) | | 11 (17.7%) | |  |
| NA | 2 (100.0%) | 1 (25.0%) | 3 (60.0%) | 27 (65.9%) | | 6 (60.0%) | | 39 (62.9%) | |  |
| **Number of times tested for COVID for any other reason** | | | | | | | | | | |
| No tests | 0 (0.0%) | 1 (25.0%) | 0 (0.0%) | 11 (26.8%) | | 2 (20.0%) | | 14 (22.6%) | |  |
| 1-2 tests | 1 (50.0%) | 2 (50.0%) | 3 (60.0%) | 17 (41.5%) | | 3 (30.0%) | | 26 (41.9%) | |  |
| 3-10 tests | 1 (50.0%) | 1 (25.0%) | 2 (40.0%) | 9 (22.0%) | | 5 (50.0%) | | 18 (29.0%) | |  |
| >10 tests | 0 (0.0%) | 0 (0.0%) | 0 (0.0%) | 2 (4.9%) | | 0 (0.0%) | | 2 (3.2%) | |  |
| Missing | 0 (0%) | 0 (0%) | 0 (0%) | 2 (4.9%) | | 0 (0%) | | 2 (3.2%) | |  |
| **Positive COVID test for any other reason** | | | | | | |  | |  | |
| Question not asked | 2 (100.0%) | 4 (100.0%) | 5 (100.0%) | 40 (97.6%) | | 8 (80.0%) | | 59 (95.2%) | |  |
| No | 0 (0.0%) | 0 (0.0%) | 0 (0.0%) | 1 (2.4%) | | 2 (20.0%) | | 3 (4.8%) | |  |
